# Oncolytic activity of Sindbis virus in human oral squamous carcinoma cells

**DOI:** 10.1038/sj.bjc.6605209

**Published:** 2009-07-28

**Authors:** K Saito, K Uzawa, A Kasamatsu, K Shinozuka, K Sakuma, M Yamatoji, M Shiiba, Y Shino, H Shirasawa, H Tanzawa

**Affiliations:** 1 Department of Molecular Virology, Graduate School of Medicine, Chiba University, 1-8-1 Inohana, Chuo-ku, Chiba 260-8670, Japan; 2 Department of Clinical Molecular Biology, Graduate School of Medicine, Chiba University, 1-8-1 Inohana, Chuo-ku, Chiba 260-8670, Japan; 3 Division of Dentistry and Oral-Maxillofacial Surgery, Chiba University Hospital, 1-8-1 Inohana, Chuo-ku, Chiba 260-8677, Japan

**Keywords:** Sindbis virus, oral squamous cell carcinoma, apoptosis

## Abstract

**Background:**

Sindbis virus (SIN) infection causes no or only mild symptoms (fever, rash, and arthralgia) in humans. However, SIN has a strong cytopathic effect (CPE) on various cancer cells. This study focuses on the oncolytic activity of SIN AR399 on oral cancer cells compared with reovirus, a well-known oncolytic virus that targets cancer cells.

**Methods:**

We analysed the cytotoxicity and growth of SIN in 13 oral squamous cell carcinoma (OSCC) cell lines (HSC-2, HSC-3, HSC-4, Ca9-22, H-1, Sa-3, KON, KOSC-2, OK-92, HO-1-N1, SCC-4, SAT, SKN-3) and normal human oral keratinocytes (NHOKs).

**Results:**

Sindbis virus infection induced CPE in 12 OSCC cell lines at a low multiplicity of infection (MOI) of 0.01, but not in the OSCC cell line, HSC-4 or NHOKs. Sindbis viral growth was not observed in NHOKs, whereas high SIN growth was observed in all OSCC cell lines, including HCS-4. The cytotoxicity and growth of SIN was the same as reovirus at an MOI of 20 in 12 OSCC cell lines. The CPE was shown, by terminal deoxyribonucleotidyl transferase–mediated dUTP nick-end labelling assays, to be apoptotic cell death. Furthermore, quantitative RT-PCR of mRNA in HSC-3 and HSC-4 cells after SIN infection showed that activation of caspases, cytochrome *c*, and I*κ*B*α* was associated with SIN-induced apoptosis.

**Conclusion:**

As a replication-competent oncolytic virus, SIN may be a useful therapeutic modality for oral cancers.

Squamous cell carcinoma (SCC) is the sixth most common cancer in the world and a major cause of morbidity (Parkin *et al*, 1999; Hunter *et al*, 2005). Treatment of oral SCC (OSCC) has primarily relied on classical therapeutic modalities including surgery, radiation, and chemotherapy or a combination of these methods; yet, the outcome of OSCC has not improved significantly. Therefore, new treatment strategies are of interest, especially gene therapy. Several gene therapy strategies for OSCC are currently under investigation in the preclinical and clinical settings (Kim *et al*, 2002; Xi and Grandis, 2003). Replication-defective viruses are used as vectors for cancer gene therapy. Several viral vector systems, such as those encoding suicide proteins, tumour suppressor proteins, or cytokines are used to introduce various genes into tumour cells. However, the efficiency of this approach is limited because vectors transduce these genes into only a small portion of the target cells. Therefore, replication-competent viruses that target cancer cells have also been studied as anti-cancer agents (Parkin *et al*, 1999; Xi and Grandis, 2003; Hunter *et al*, 2005).

Sindbis virus (SIN) is an RNA virus belonging to the *Alphavirus* genus in the *Togaviridae* virus family; it is transmitted to birds and mammals by mosquito bites (Jan and Griffin, 1999) and subsequently spreads throughout the body through the bloodstream (Tseng *et al*, 2002; Unno *et al*, 2005). It has the potential to induce apoptosis of infected mammalian cells (Jan and Griffin, 1999; Moriishi *et al*, 2002). In addition, one of the surface receptors on mammalian cells mediating SIN infection has been identified as the 67-kDa, high-affinity laminin receptor (Wang *et al*, 1992), reported to be highly expressed in various human cancers (van den Brule *et al*, 1996). Sindbis virus infection induces no or only mild symptoms (fever, rash, and arthralgia) in humans (Jan and Griffin, 1999). Several replication-defective SIN vector systems have been studied for *in vitro* gene transfer into mammalian cells and *in vivo* gene therapy (Tseng *et al*, 2002; Yamanaka, 2004; Unno *et al*, 2005). However, whether replication-competent wild-type SIN can be used as an anti-cancer agent in oral cancers has not been investigated.

In this study, we tested 13 OSCC cell lines, as well as normal human oral keratinocytes (NHOKs), for susceptibility to SIN infection *in vitro*. For comparison, cell lines were also infected with reovirus, a well-known oncolytic virus with Ras pathway activation (Hirasawa *et al*, 2002). We also evaluated the expression of genes involved in apoptosis after SIN infection.

## Material and methods

### Cell lines and cell culture

The OSCC-derived cell lines HSC-2, HSC-3, HSC-4, SCC-4, SAT, Ca9-22, SKN-3, KON, HO-1-N1, KOSC-2 (Human Science Research Resources Bank, Osaka, Japan), OK-92 (established from a carcinoma of the tongue in our department), Sa-3, and H-1 (provided by Dr Shigeyuki Fujita, Wakayama Medical University, Wakayama, Japan) were grown in Dulbecco's modified Eagle's medium (DMEM) with 10% fetal bovine serum (FBS) and 50 units per ml of penicillin and streptomycin. Laboratory stock Vero cells were maintained in DMEM supplemented with 10% FBS at 37°C in 5% CO_2_. Primary cultured NHOKs were grown in KGM.

### Viruses

The SIN AR339 (wild-type) virus used in this study was provided by the National Institute of Infectious Diseases (Tokyo, Japan). The virus was propagated in primary chicken embryo fibroblast cells, then passed several times in Vero cells and used as a laboratory stock. AR339 was propagated in C33A cells maintained in DMEM supplemented with 10% FBS at 37°C in 5% CO_2_. Viral titres were determined by plaque assays using monolayers of Vero cells. The laboratory stock reovirus strain T3D used in this study was propagated in L cells (ATCC, Manassas, VA, USA), and viral titres were determined by plaque assays using monolayers of L cells.

### Cytotoxicity of viruses in cell lines

Cells were plated onto 24-well plates at 1 × 10^5^ cells per well and infected with SIN at a multiplicity of infection (MOI) of 20, 1, 0.1, or 0.01, or reovirus at an MOI of 20. After incubation for 96 h, cell viability was evaluated and the medium was removed. Each plate was equilibrated to room temperature (about 25°C) for 20 min, followed by addition of 200 *μ*l of CellTiter Glo reagent (Promega, Madison, WI, USA) to each well. Plates were gently shaken on a plate shaker for 2 min, incubated for an additional 10 min at room temperature after which cell viability was evaluated immediately by luminescence measured with a plate reader (Wallac 1420 ARVOsx Multilabel Counter; PerkinElmer, Chiba, Japan). Cell viability of mock-infected cells was designated as 100% viability. Data are presented as the mean±s.d. of three determinations.

### Viral growth in cells

Cells grown in 24-well plates were infected with SIN or reovirus at an MOI of 0.1. After incubation for 96 h, the culture medium was collected. After freezing and thawing once, the viral titres were determined by plaque assays using Vero cells.

### Western blot analysis

Cells were lysed in buffer (10 mM Tris base (pH 8.0), 400 mM NaCl, 3 mM MgCl_2_, 0.5% Nonidet P-40 (Sigma, St Louis, MO, USA), 100 mM phenylmethylsulfonyl fluoride, and 0.01% protease inhibitor cocktail (Sigma) at 4°C for 10 min. Protein extracts were electrophoresed on 10% sodium dodecyl sulphate-polyacrylamide electrophoresis gels (SDS-PAGE) and transferred to polyvinylidene fluoride (PVDF) membranes (Bio-Rad, Hercules, CA, USA). After transfer, the PVDF membranes were washed with 0.1% Tween 20 in TBS, and incubated with the primary antibody (rabbit anti-SIN polyclonal antibody, 1 : 500; Laminin-R (Santa Cruz, Heidelberg, Germany) 1 : 5000 overnight at 4°C for immunoblotting. Then, PVDF membranes were washed again and incubated with a 1 : 2000 dilution of horseradish-peroxidase-conjugated IgG Envision+(Dako, Carpinteria, CA, USA), as a secondary antibody, for 2 h at room temperature. Finally, the membranes were incubated with enhanced chemiluminescence (ECL)+ horseradish peroxidase substrate solution included in the ECL+kit (GE Healthcare UK Ltd, Chalfont St Giles, UK). Protein bands were visualised by exposing the membrane to Hyperfilm (GE Healthcare UK Ltd).

### Ras activation assay

Confluent cells (70–80%) grown in 10-cm dishes were lysed with 1 × Mg^2+^ lysis buffer (Ras activation assay kit; Upstate Biotechnology, Lake Placid, NY, USA). To determine the level of activated Ras (Ras-GTP) in these cells, we incubated 1 mg of cell lysate with 10 *μ*l of Raf-1 Ras binding domain agarose conjugate at 4°C for 30 min. The beads were then collected, washed, re-suspended in 2 × Laemmli buffer, and boiled for 5 min. This was then followed by SDS-PAGE and western blotting with an anti-Ras antibody (clone RAS 10) according to the manufacturer's instructions. To determine the level of total Ras, we directly subjected cell lysates to SDS-PAGE and western blotting with anti-Ras antibody.

### Terminal deoxyribonucleotidyl transferase–mediated dUTP nick-end labelling (TUNEL) assays

A total of 5.0 × 10^5^ cells seeded on Lab-Tek chamber slides (Nalge Nunc International, Rochester, NY, USA) were incubated in the presence or absence of SIN at an MOI of 1 for 24 h. The cells on chamber slides were washed twice with PBS, air dried, and fixed with 4% paraformaldehyde at room temperature for 30 min. The TUNEL assays were carried out by using an *in situ* apoptosis detection kit (TaKaRa, Tokyo, Japan) according to the manufacturer's instructions. Cells were viewed and photographed under a fluorescence microscope (Nikon Inc., Tokyo, Japan).

### Real-time RT-PCR analysis of gene expression

Total RNA was isolated from cells using Trizol reagent (Invitrogen, Carlsbad, CA, USA) according to the manufacturer's protocol. Double-stranded cDNA was synthesised from 20 *μ*g of total RNA using Ready-to-Go You-Prime first-strand beads (GE Healthcare UK Ltd) and Oligo(dT) primer (Sigma-Genosys, Ishikari, Japan). Real-time quantitative RT-PCR was performed with a single method using a LightCycler FastStart DNA Master SYBR Green 1 Kit (Roche Diagnostics GmbH, Mannheim, Germany). We chose to assess the expression of 15 genes (Table 1) related to apoptotic pathways. The transcript levels of these genes were estimated from the respective standard curves and normalised to the expression of glyceraldehyde-3-phosphate dehydrogenase (GAPDH) (forward, 5′-CATCTCTGCCCCCTCTGCTGA-3′ and reverse, 5′-GGATGACCTTGCCCACAGCCT-3′) transcript levels determined in corresponding samples. The experiments were carried out in triplicate.

## Results

### Cytopathic effects (CPEs) of SIN and reovirus

**Figure 1:**
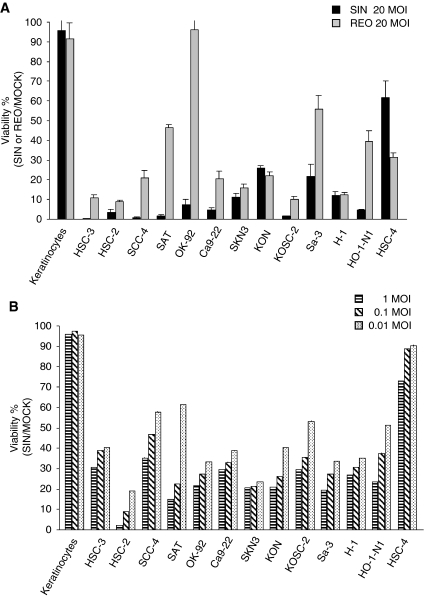
Cell viability assays of cells infected with SIN or reovirus. (**A**) Cells, 1 × 10^4^, were plated and mock-infected or infected with SIN or reovirus at an MOI of 20 for 96 h. The surviving cells were evaluated by using the luminescence with a plate reader. Cell viability of mock-infected cells was designated as 100% viability. (**B**) Cell viability assays of cells infected with SIN. Cells, 1 × 10^4^, were plated and mock-infected or infected with SIN at the indicated MOI. The surviving cells were evaluated by luminescence on a plate reader 96 h after infection. Cell viability of mock-infected cells was designated as 100% viability.

To determine the susceptibility of OSCC to SIN, we carried out cell viability assays. The OSCC cell lines (HSC-2, HSC-3, HSC-4, SCC-4, SAT, Ca9-22, SKN-3, KON, HO-1-N1, KOSC-2, OK-92, Sa-3, and H-1), as well as primary cultured keratinocytes derived from normal oral tissues, were infected with SIN at an MOI of 20, 1, 0.1, or 0.01. For comparison, cells were also infected with reovirus at an MOI of 20. As shown in Figure 1, 96 h after SIN infection at an MOI of 20, strong CPEs were observed in all OSCC cell lines except HSC-4 (Figure 1). No morphologic change was observed in normal keratinocytes, whereas reovirus infection caused similar CPEs in 10 cell lines as well as in normal cells. The CPEs were weaker in three cell lines (SAT, OK-92, Sa-3, HO-N-1), and stronger in HSC-4. Sindbis virus infection at an MOI of 1, 0.1, or 0.01 also induced sufficient CPEs in all OSCC cell lines except HSC-4.

### Viral growth of SIN in cancer cells

**Figure 2:**
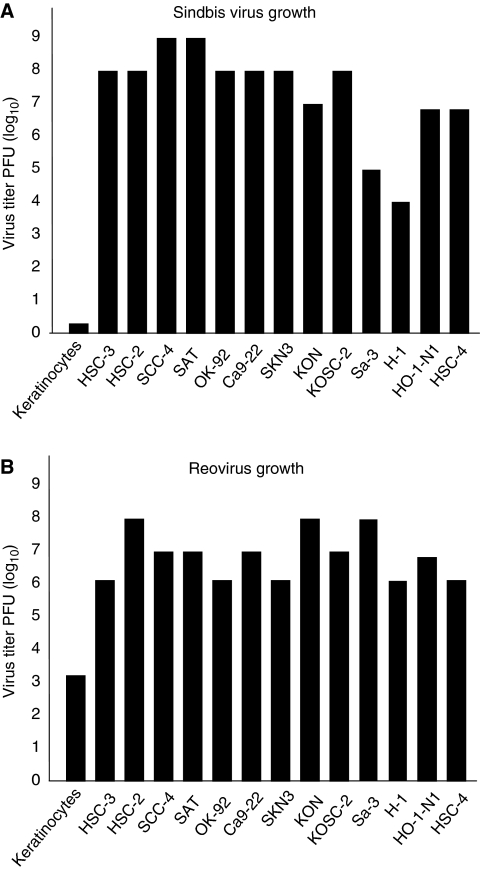
Viral growth (**A** and **B**) in cells infected with SIN. (**A**) Titre of virus grown in various cells (keratinocytes, HSC-2, HSC-3, SCC-4, SAT, OK-92, Ca9-22, SKN-3, KON, KOSC-2, Sa-3, H-1, HO-1-N1, and HSC-4) was measured 96 h after SIN infection at an MOI of 0.1. (**B**) The titre of virus in cells (keratinocytes, HSC-2, HSC-3, SCC-4, SAT, OK-92, Ca9-22, SKN-3, KON, KOSC-2, Sa-3, H-1, HO-1-N1, and HSC-4) was measured 96 h after reovirus infection at an MOI of 0.1.

To examine whether the extent of cell cytotoxicity after SIN infection correlates with viral growth, viral titres in the cancer cell lines were measured 96 h after SIN or reovirus infection (0.1 MOI). High growth of SIN and reovirus was observed in all OSCC cell lines (Figure 2) whereas no viral growth was observed in normal keratinocytes, supporting the idea that the cytotoxicity caused by SIN depends on replication of the virus. However, there was little correlation between the viral titre and cytotoxicity caused by infection. For example, the SIN viral growth was high in HSC-4 cells but SIN infection caused little cytotoxicity in these cells. In contrast, viral growth was low in Sa-3 and H-1 cells but SIN caused high cytotoxicity in these cell lines.

### Expression of SIN protein

**Figure 3:**
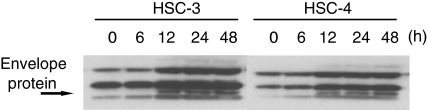
Western blot analysis of SIN proteins, E1 and E2. SIN protein synthesis in cells was analysed 6, 12, 24, and 48 h after SIN infection at an MOI of 1 in HSC-3 and HSC-4 cells. An arrow indicates the position of the SIN E1 and E2 proteins.

To further determine whether the cytotoxicity caused by SIN infection correlates with SIN replication, the SIN protein synthesis in cells was analysed 6, 12, 24, and 48 h after SIN infection at an MOI of 1 in HSC-3 and HSC-4 cells. The reason two cell lines were chosen was that the HSC-4 cells displayed the lowest levels of CPE and the HSC-3 cells displayed the highest levels of CPE at an MOI of 20 (Figure 1A). As shown in Figure 3, immunoblot analyses with anti-SIN antibody showed that SIN protein synthesis in both HSC-3 and HSC-4 was substantial 12 h after SIN infection. High viral growth and viral protein expression in HSC-4 cells occurred, but little cytotoxicity was observed after infection with SIN, suggesting that the cytotoxicity caused by SIN infection is not the direct result of viral protein synthesis and viral replication.

### Ras activities in cancer cells

**Figure 4:**
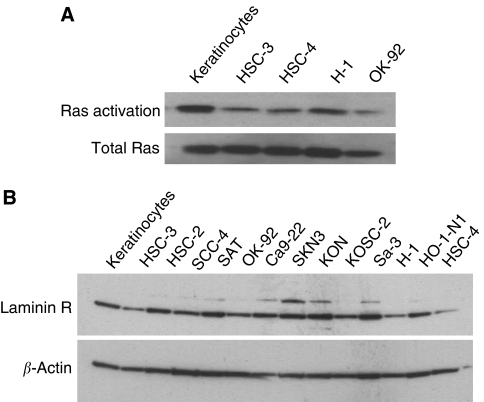
(**A**) The activated Ras protein levels in cancer cell lines (HSC-3, SCC-4, H-1, OK-92) and normal human keratinocytes (keratinocytes) were examined. The amount of Ras-GTP was measured using Raf-1-Ras binding domain conjugated to agarose beads to pull down activated Ras. This was then followed by SDS-PAGE and western blotting with an anti-Ras antibody. (**B**) The expression of 67-kDa laminin receptor in all cancer cell lines and normal cells was examined using SDS-PAGE and western blotting with a Laminin-R antibody.

The GTP-bound, active form of Ras stimulates the downstream effector, Raf-1 through its binding to the Raf-1-Ras binding domain. To examine the activation of Ras in cancer cell lines, we measured the amount of Ras-GTP using Raf-1-Ras binding domain conjugated to agarose beads (Upstate Biotechnology) to pull down activated Ras. Lysates were prepared from cells grown to 80% confluence under normal growth conditions. As shown in Figure 4A, elevated levels of Ras activity were observed in both normal human keratinocytes and cancer cell lines except OK-92, which was shown not to be susceptible to reovirus (Figure 1A). Apoptosis after reovirus infection is Ras dependent.

### Expression of laminin-R in cancer cells

It has been reported that a 67-kDa laminin receptor, which is substantially upregulated in numerous cancers, is a major receptor of SIN in mammalian cells. We examined the expression of the 67-kDa laminin receptor in all cancer cell lines and normal human keratinocytes. We confirmed that the 67-kDa laminin receptor is expressed in normal human keratinocytes as well as our cancer cell lines, except for in HSC-4 cells (Figure 4B).

### SIN-induced apoptosis in cancer cell lines

**Figure 5:**
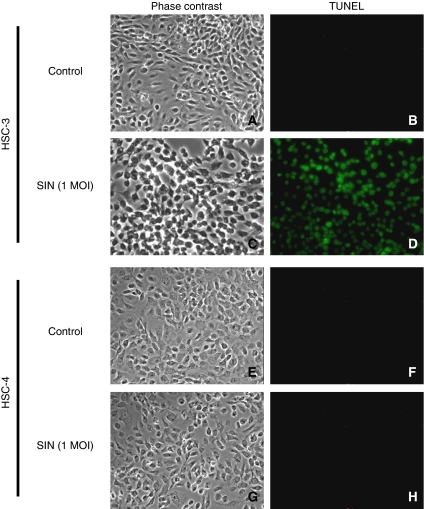
TUNEL assays of cells infected with SIN. HSC-3 (**A**–**D**) and HSC-4 (**E**–**H**) were infected with SIN (phase contrast, **B** and **F**; TUNEL, **D** and **H**) at an MOI of 1, and without SIN (phase contrast, **A** and **E**; TUNEL, **C** and **G**).

Viral infection often leads to an apoptotic response in infected cells. To determine whether infection with SIN induces apoptosis in HSC-3 and HSC-4, we carried out TUNEL assays. We observed TUNEL-positive cells in HSC-3 cells 24 h after SIN infection at an MOI of 1 (Figure 5). In contrast, few apoptotic cells were observed in HSC4-cells, indicating that the cytotoxicity of SIN infection correlates with the apoptosis induced by viral infection, but not always with the extent of viral replication.

### Apoptotic pathway

**Figure 6:**
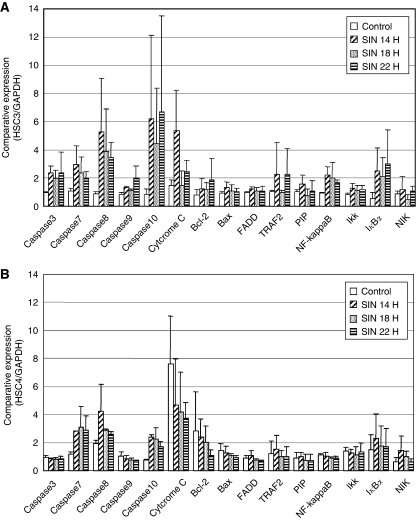
Expression of apoptosis-related genes after SIN infection. The expression of mRNAs related to apoptosis in (**A**) HSC-3 and (**B**) HSC-4 cells was measured in triplicate using quantitative PCR 6, 12, and 18 h after SIN infection at an MOI of 1. All bars represent average values±standard deviation from triplicate samples, which were normalised to GAPDH expression.

To determine which apoptotic pathway is induced by SIN, we examined the expression of mRNAs relating to apoptosis in HSC-3 and HSC-4 cells by quantitative PCR 14, 18, and 22 h after SIN infection at an MOI of 1. Caspases 7, 8, and 10 were upregulated in both HSC-3 and HSC-4 cells. However, caspases 3 and 9, cytochrome *c*, NF-*κ*B, and IKK were remarkably upregulated only in HSC-3 cells (Figure 6).

## Discussion

In this study, we investigated whether SIN causes cytotoxic effects in OSCC cells. We demonstrated that SIN can induce more cell death than reovirus at an MOI of 20 in 10 of 13 OSCC cell lines. Furthermore, all OSCC cell lines, with one exception, were susceptible to SIN infection at a low MOI. Viral growth assays with OSCC cell lines indicated that SIN can grow to a high titre, and that cytotoxicity does not always correlate with the extent of viral growth and replication. In addition, normal keratinocytes are not susceptible to SIN, and did not support viral growth and replication.

Although it has been reported that the specific cytotoxicity induced by SIN infection in cancer cells was due to the expression of the 67-kDa laminin receptor on the cell membrane, we confirmed that normal keratinocytes had the same level of expression of the 67-kDa laminin receptor as cancer cells, with the exception of HSC-4. It was suggested that there is a difference in occupancy of laminin receptors in normal tissues compared with tumour tissues, and that this could provide an explanation for the natural propensity of SIN towards tumour cells (Hay, 2004). The discrepancy between the high replication and low expression of laminin receptors in HSC-4 might be because SIN can use receptors other than the laminin receptor to enter mammalian cells. These results could be responsible for the correlation with cytotoxicity and viral replication in normal human keratinocytes and OSCC cell lines but not in HSC-4. We next investigated why replication of SIN in HSC-4 induced little cytotoxicity. Reovirus is a novel oncolytic agent for cancer therapy based on its targeting of the activated Ras signalling pathway. Roughly 50% of all cancers have an activated Ras signalling pathway because of activating mutations in the *ras* gene itself, and of genes for proteins that act up- or downstream of *ras*. Therefore, we evaluated whether the oncolytic potency of SIN in oral cancer cells depends on increased Ras activity in these cells. In cancer cells including HSC-4, *ras* activation was correlated with the CPE of reovirus, but not SIN. In particular, OK-92 had a strong CPE after SIN infection, but Ras activation was very weak in this cell line. A number of studies suggest that apoptosis is important in viral CPEs and the mechanism of SIN-induced apoptosis may be related to members of the caspase family and Bcl-2 proteins (Nava *et al*, 1998; Kerr *et al*, 2002). Our study showed that cell death in HSC-3 cells, the most susceptible of the cell lines to SIN, was induced by apoptosis. Therefore, we investigated which apoptotic pathway was activated in HSC-3 and HSC-4 cells and compared the levels of gene expression of all known apoptotic mediators. In HSC-3 cells, members of the caspase family, cytochrome *c*, and I*κ*B*α* were upregulated, indicating that SIN causes oncolysis by activating several apoptotic pathways. The upregulation of Bcl-2 and NF-*κ*B, known anti-apoptotic factors which may be involved in cell survival, was not observed in HSC-4 cells. Consequently, these findings suggest that another mechanism that inhibits cell death may be activated in HSC-4 cells.

The observation that SIN could replicate but could not induce apoptosis in the HSC-4 cell line suggests that the link between apoptosis and viral replication does not exist in these cells. Vertebrate cell death caused by SIN infection was shown to be caused by apoptosis and resulted in a lytic infection. In contrast, all invertebrate cells tested to date survive infection, with some cultures displaying varying degrees of CPE and becoming persistently infected for long periods of time (Karpf *et al*, 1997). Viral growth in invertebrates has been studied in various species of mosquitoes (Greene *et al*, 2005; Mudiganti *et al*, 2006). Invertebrate cells are known to differ from mammalian cells during SIN infection in that SIN replicates and assembles in these cells without causing apoptosis (Miller and Brown, 1992), but the processes involved are not clearly understood. Given that HSC-4 cells showed only slight CPEs during the acute phase of infection, as was observed in invertebrate cells (data not shown), the same mechanism may be involved in HSC-4 cells. Our study suggests that an apoptotic pathway, possibly related to caspases 3 and 9, cytochrome *c*, NF-*κ*B, I*κ*B*α*, and IKK, might be involved in the cytotoxicity caused by SIN infection.

The most favourable feature of SIN is that it can attach and enter all OSCC cell lines but not normal keratinocytes, a characteristic that would be useful for gene therapy in oral cancer. The goal of cancer gene therapy is to introduce new genetic material into target cells without damaging normal tissues. Viruses commonly used in oral cancer gene therapies include retroviruses and adenoviruses. Transduction studies have demonstrated that direct injection, but not topical application, of adenoviral constructs can transduce genes into oral cancer cells *in vivo* (Clayman *et al*, 1995). These therapies are limited by safety and targeting to tumours.

Targeting is a key determinant of the safety of viral vectors. As the dose of a viral vector increases, improvement may be seen, but side effects can also increase. Therefore, an effective, low-dose treatment is necessary to target cancer cells. Recently, a replication-defective SIN viral vector was developed as a gene therapy vector for transient and high-level expression of heterologous genes (Tseng *et al*, 2004). However, replication-competent wild-type SIN has an additional favourable feature for a modality of cancer therapy (Unno *et al*, 2005). Replication-competent oncolytic viruses, like SIN used in this study, would not follow a simple dose–response curve. Theoretically, a single viable virus particle could potentially kill an entire tumour if it replicates and spreads efficiently throughout the tumour mass.

In conclusion, SIN appears to be a feasible therapeutic modality for targeting oral cancer cells. With further work, SIN could be a useful oncolytic viral therapy in oral cancers.

## Figures and Tables

**Table 1:** Primer sequences for apoptosis-related genes

**Name**	**Forward**	**Reverse**
*Caspase 3*	TGAGCCTGAGCAGAGACATGA	CCTTCCTGCGTGGTCCAT
*Caspase 7*	AAGCTGGGCAAATGCATCAT	TGTATGGTCCTCTTCAGAAGCTTTT
*Caspase 8*	GGTGGCTGCCTGAGGAATAC	TCCCAAGGTTCAAGTGACCAA
*Caspase 9*	CAGTAACCCCGAGCCAGATG	TGAGCCCACTGCTCAAAGATG
*Caspase 10*	AGTGGACAAACAGGGAACAAAGA	GGTTATAGCCAATGATTCGTTTGA
*Bcl-2*	CATGTGTGTGGAGAGCGTCAA	GCCGGTTCAGGTACTCAGTCA
Cytochrome *c*	TGGTTGCACTTACACCGGTACT	ACGTCCCCACTCTCTAAGTCCAA
*Bax*	TTGCCGTCAAAACATGTCA	CCGCCGTGGACACAGACT
*FADD*	CCTGCACAGATATTTCCATTTCTTC	GTGGAGTAACAGTGTGACTGCTCAT
*PIP1*	TGGTACAGGCCAATTCCAAGT	TGATGAACTCGTCCCACCAA
*TRAF2*	TCAGGACCACGTCAAGACTTGT	CAGCACCGAGCTCAGTAGCA
*NIK*	GTTCAGCCCCACCTTTTCAG	TTTGCTGCGACGCTTTCC
*IKK*	GCAGGCTCTTTCAGGGACAA	TCCAAGTCAAGCTGAATGCTTTT
*IκBα*	TCCTGCACTTGGCCATCAT	GCAATTTCTGGCTGGTTGG
*NF-*κ*B*	AATGGGCTACACCGAAGCAA	GCCGCTGTCGCAGACACT
